# Extrahepatic Portal Vein Occlusion and Collateral Formation Following Anticoagulant-Induced Thrombosis Regression in a Portal Vein Aneurysm: Potential for Misdiagnosis As Primary Extrahepatic Portal Vein Occlusion

**DOI:** 10.7759/cureus.92610

**Published:** 2025-09-18

**Authors:** Hiroshi Okano, Satomi Tsuruga, Katsumi Mukai, Akira Nishimura

**Affiliations:** 1 Gastroenterology, Suzuka General Hospital, Suzuka, JPN

**Keywords:** anticoagulant, extrahepatic portal obstruction, primary extrahepatic portal vein aneurysm, secondary eho, thrombosis

## Abstract

Both extrahepatic portal obstruction (EHO) and primary extrahepatic portal vein aneurysm (PVA) are rare diseases of the hepatic vascular system with limited understanding of their natural histories. While there is no known relationship between the onset or formation of these two conditions, we encountered a case that may suggest a mutual association. A 40-year-old man was presented to the Department of Gastroenterology at our hospital with complaints of dull abdominal pain and fever. Three years prior, the patient had been incidentally diagnosed with a primary extrahepatic PVA. Due to an inflammatory reaction and elevated hepatic enzymes shown in laboratory data, along with a thrombosis complicating the PVA, anticoagulant therapy was initiated. Following the introduction of this treatment, his abdominal symptoms and inflammatory reaction improved. Subsequent follow-up abdominal imaging showed that both the PVA thrombosis and the aneurysm itself had diminished. The patient's portal vein system ultimately developed EHO with collateral vessels. This case is significant because it suggests a potential etiology for what is conventionally diagnosed as "idiopathic" EHO in adults. It highlights the importance of cautiously diagnosing EHO, considering the possibility that it may develop as a complication of portal vein obstruction caused by PVA thrombosis.

## Introduction

Extrahepatic portal obstruction (EHO) is a rare disease caused by obstruction of the extrahepatic portal vein, including the porta hepatis [1]. Apart from secondary EHO cases due to surrounding organ diseases, such as pancreatic carcinoma or pancreatitis [2], the etiology of primary EHO remains unknown. However, a congenital or hereditary origin is suggested for EHO due to its characteristic age distribution, which shows the most common diagnostic age to be under 10 years [1]. While many cases are diagnosed in childhood, the diagnostic age of EHO also shows another distribution, with cases in patients in their 40s and 50s [1]. It is unknown whether EHO diagnosed in middle-aged patients is due to congenital or hereditary factors.

Primary extrahepatic portal vein aneurysm (PVA) is also a rare visceral venous aneurysm, and its etiology also remains unclear [3]. Because PVA is often observed in cases of cirrhosis and portal hypertension, PVA cases associated with these conditions are thought to be acquired [4,5]. On the other hand, similar to EHO, PVA is found in children and young adults without portal hypertension [4]. The presence of non-cirrhotic and younger PVA cases suggests that primary PVA, without cirrhosis and portal hypertension, is caused by a congenital disorder. Irrespective of whether the case is primary or acquired, the most common complications of PVA are thrombosis and rupture [4,5]. However, many cases of PVA may exist unnoticed, as most PVA cases occur without complications [4,6]. Furthermore, most PVA cases are incidentally discovered by imaging modalities targeting other organs [5].

In this article, we report a case of PVA. This patient's PVA was detected incidentally during an abdominal examination, and the patient subsequently developed portal vein thrombosis within the aneurysmal space. The thrombosis diminished after the introduction of anti-coagulation treatment. Interestingly, the diminishing portal vein thrombosis was concomitant with the shrinking and diminishing of the PVA, the emergence of EHO at the former PVA position, and the appearance of collateral vessels around the porta hepatis.

## Case presentation

**Figure 1:**
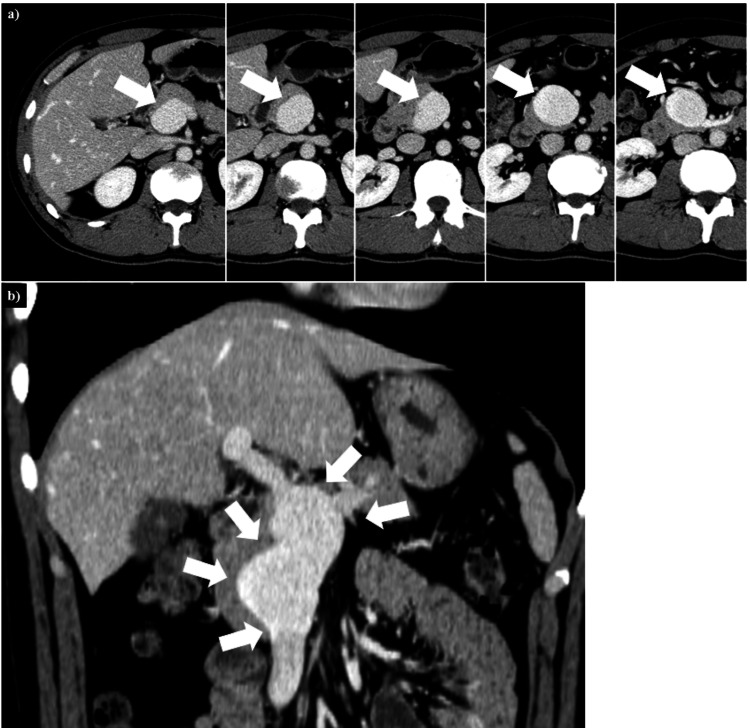
Enhanced CT images of a PVA from three years prior (a) Transverse images of the PVA. This series of images was captured from rostral (left) to caudal (right). The portal vein was enlarged, measuring 36 mm in diameter (white arrows). No thrombosis was detected. (b) Coronal images of the PVA. The PVA extended from the main portal vein trunk to beyond the confluence of the splenic and superior mesenteric veins (white arrows). CT: computed tomography, PVA: portal vein aneurysm

A 40-year-old man was presented to the Department of Gastroenterology at our hospital complaining of dull abdominal pain and fever. His abdominal pain was located around the epigastric region, and he had mild tenderness but no muscle guarding. He had been experiencing abdominal pain for about three weeks, along with a fever that lasted three days. Additionally, he had a daily habit of drinking 700 ml of beer. Three years ago, he incidentally had a PVA detected by abdominal CT imaging (Figure 1).

The PVA was 36 mm in diameter and fusiform. Due to the absence of complications, such as thrombosis, there was no medication. The patient was not followed up for the PVA because he declined the follow-up visit. Apart from the PVA detection, the patient developed an allergic reaction with a skin rash after using contrast enhancement materials for CT imaging, and the patient was subsequently prohibited from using CT-associated contrast enhancement materials.

Table 1 summarizes the laboratory findings at presentation with abdominal symptoms. Liver-associated enzymes, specifically aspartate aminotransferase (AST), alanine aminotransferase (ALT), lactate dehydrogenase, alkaline phosphatase, and γ-glutamyltransferase, exhibited mild elevations. Although an inflammatory disorder was suspected, given the elevated C-reactive protein (CRP) and procalcitonin levels, the white blood cell (WBC) count remained within the normal range. Coagulation analysis demonstrated elevated levels of fibrinogen, fibrin/fibrinogen degradation products (FDP), and D-dimer.

**Table 1:** Laboratory data of the present case CBC: complete blood count, WBC: white blood cells, RBC: red blood cells, PT: prothrombin time, PT-INR: prothrombin time-international normalized ratio, APTT: activated partial thromboplastin time, FDP: fibrin/fibrinogen degradation products, ATIII: antithrombin III, AST: aspartate aminotransferase, ALT: alanine aminotransferase, LDH: lactate dehydrogenase, ALP: alkaline phosphatase, g-GT: g-glutamyltransferase, T-Bil: total bilirubin, D-Bil: direct bilirubin, FBS: fast blood glucose, T-chol: total cholesterol, BUN: blood urea nitrogen, eGFR: estimated glomerular filtration rate, CRP: C-reactive protein, Ig: immunoglobulin ^#1 ^Serum ALP levels measured using the International Federation of Clinical Chemistry and Laboratory Medicine (IFCC) method could be calculated as 0.34 times the ALP levels measured using the Japan Society of Clinical Chemistry (JSCC) method.

Lab parameters	Reference	At the onset of portal vein thrombosis
CBC		
WBC (/mL)	3900-9800	5600
RBC (/mL)	376-500	403 × 10^4^
Hemoglobin (g/dL)	11.3-15.2	11.8
Hematocrit (%)	33.4-44.9	36.4
Platelets (/mL)	130-369	28.0 × 10^4^
Coagulation		
PT (%)	70-130	68
PT-INR	0.8-1.2	1.20
APTT (/second)	25-45	35.5
Fibrinogen (mg/dL)	200-400	639
FDP (ug/mL)	0-5	7.7
D-dimer (ug/mL)	0-1	3.7
ATIII (%)	79-121	100
Chemistry		
AST (IU/L)	10-35	45
ALT (IU/L)	10-35	77
LDH (IU/L)	110-225	271
ALP (IU/L)	72-113	^#1^172
g-GT (IU/L)	8-60	76
T-Bil (mg/dL)	0.2-1.3	0.5
D-Bil (mg/dL)	0.1-0.5	0.1
FBS (mg/dL)	70-109	96
T-chol (mg/dL)	150-219	168
BUN (mg/dL)	9.6-22	12.1
Creatinine (mg/dL)	0.5-1.1	0.67
Na (meq/L)	138-145	144
K (meq/L)	3.4-4.7	4.5
Cl (meq/L)	99-108	106
eGFR (mL/min/1.73m2)		104.30
CRP (mg/dL)	0-0.3	13.33
Procalcitonin (ng/mL)	0-0.05	0.12
IgG (mg/dL)	870- 1700	1204
IgA (mg/dL)	110-410	210
IgM (mg/dL)	35-220	123

**Figure 2:**
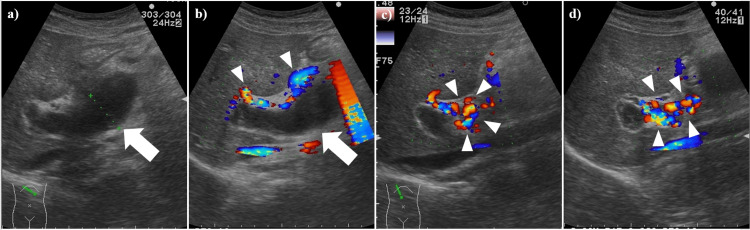
Abdominal US images of a PVA at the presentation of abdominal symptoms (a) B-mode US demonstrated a dilated portal vein (white arrow). (b-d) Doppler mode revealed an absence of blood flow within the dilated portal vein trunk (white arrow); however, collateral vessels exhibiting blood flow were observed surrounding the trunk (white arrowheads). US: ultrasonography, PVA: portal vein aneurysm

Abdominal ultrasonography was performed on the patient to investigate abdominal pain, fever, and abnormal laboratory findings. The ultrasonography imaging revealed a dilated portal vein (Figure 2).

**Figure 3:**
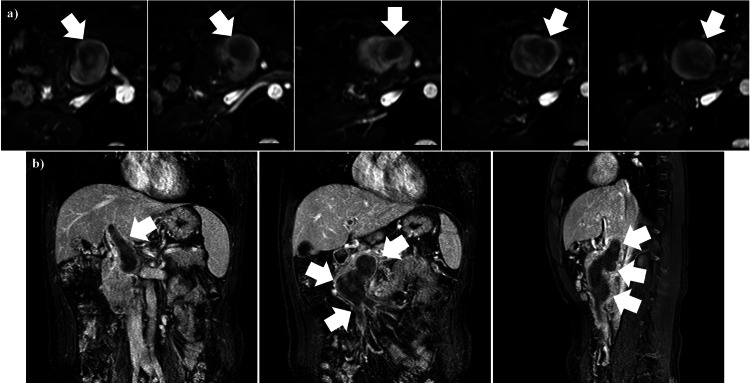
Abdominal MRI with Gd-EOB-DTPA (a) Transverse images of the PVA. This series of images was captured from rostral (left) to caudal (right). The PVA lumen showed a non-blood flow area, indicated by the non-enhancement area occupying the PVA lumen (white arrows). (b) Coronal (left and center) and sagittal (right) images of the PVA and portal vein system. The non-enhancement area extended from the intra-hepatic portal vein through the portal vein trunk and PVA to the superior mesenteric vein (white arrows). MRI: magnetic resonance imaging, Gd-EOB-DTPA: gadolinium-ethoxybenzyl-diethylene-triamine-pentaacetic acid, PVA: portal vein aneurysm

No blood flow was detected within the dilated portal vein trunk; however, the imaging demonstrated collateral vessels with blood flow surrounding the trunk. For malignancy surveillance, the patient underwent esophagogastroduodenoscopy (EGD). No significant diseases were detected, except mild esophageal varices, which were likely attributed to portal hypertension resulting from blood flow disturbance within the portal vein trunk (data not shown). Furthermore, the patient's chest CT (data not shown) and abdominal MRI with gadolinium-ethoxybenzyl-diethylene-triamine-pentaacetic acid (Gd-EOB-DTPA) revealed no malignant regions. On the Gd-EOB-DTPA MRI, both the portal vein system and the PVA showed extensive non-enhancement in their lumens, extending from the intrahepatic portal vein through the portal vein trunk and PVA to the superior mesenteric vein (Figure 3).

**Figure 4:**
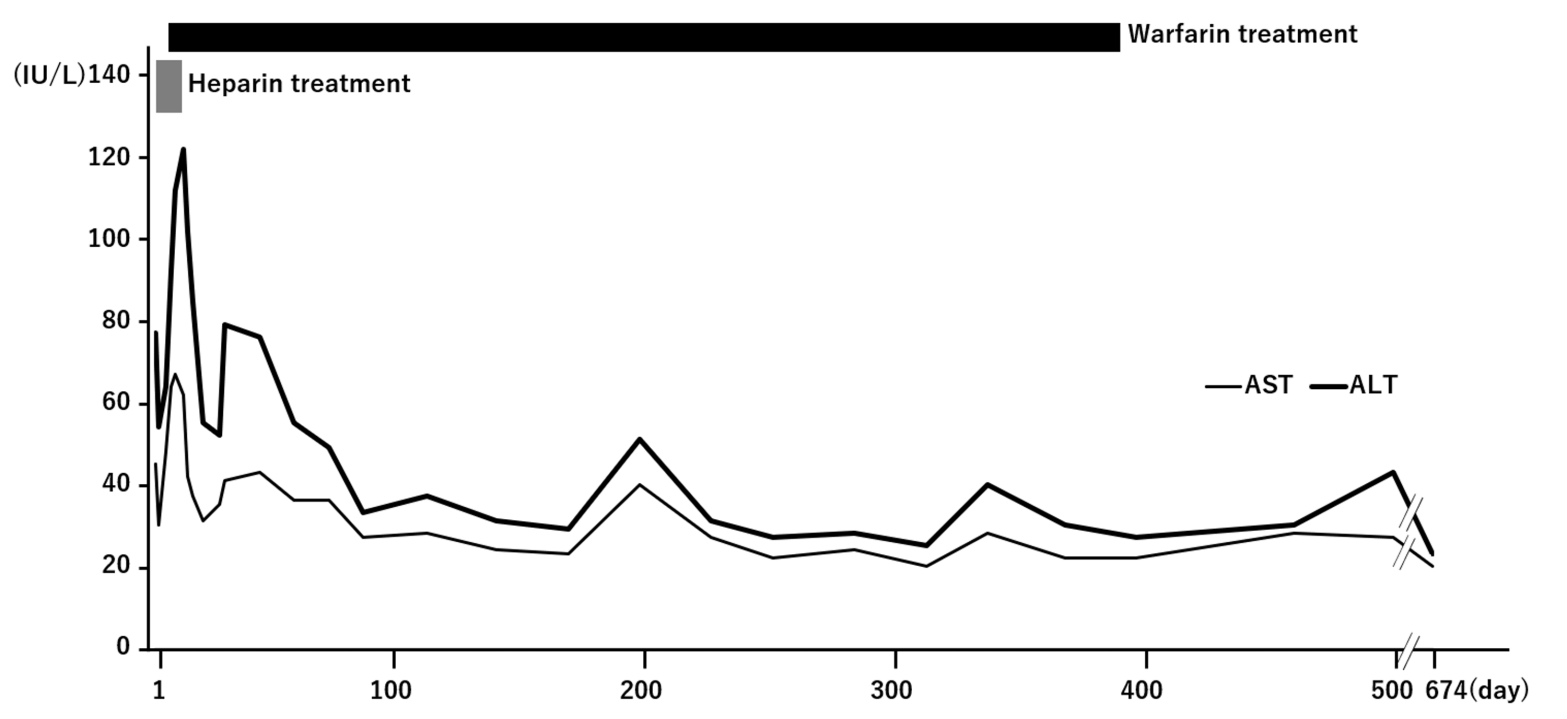
Clinical course of PVA thrombosis The gray bar indicates the period of heparin treatment, and the black bar indicates the period of warfarin treatment. AST and ALT levels decreased after the initiation of anticoagulant treatments. PVA: portal vein aneurysm, AST: aspartate aminotransferase, ALT: alanine aminotransferase

Given the Gd-EOB-DTPA MRI findings of intraluminal material within the portal vein system (including the PVA), the lack of detected malignancy, and laboratory results suggestive of non-infectious inflammation with elevated FDP/D-dimer, we diagnosed thrombosis affecting the PVA and its associated portal venous system, specifically involving the intrahepatic portal vein and superior mesenteric vein. Following the diagnosis of PVA with thrombosis, anticoagulant treatment with heparin was initiated (Figure 4).

Prior to initiating anticoagulant treatment for PVA thrombosis, we measured various coagulation-associated factors in the patients' serum to assess for inherent or acquired coagulopathy (Table 2).

**Table 2:** Laboratory data of the present case (2) HBsAg: hepatitis B surface antigen, COI: cut-off index, Ab: antibody, HCV: hepatitis C virus, HIV: human immunodeficiency virus, RPR: rapid plasma reagin, R.U.: RPR units, TPLA: treponema pallidum latex agglutination, ANA: antinuclear antibody

Lab parameters	Reference	At the onset of portal vein thrombosis
Viral and infection marker		
HbsAg (COI)	0-0.99	0.1
HCVAb (COI)	0-0.9	0.1
HIVAg		(-)
RPR (R.U.)	0-0.9	0.2
TPLA (COI)		0.0
Serology		
ANA	0-39	<40
Anti-DNA Ab (IU/mL)	0-6	3.0
Lupus anticoagulant	0-6.3	1.1
Von Willebrand factor Ag (%)	50-150	>201
Plasminogen (%)	71-128	118
Fibrin monomer (mg/mL)	0-6.1	<3.0
Alpha2-plasmin inhibitor plasmin complex (mg/mL)	0-1.7	2.5
Thrombin-antithrombin complex (ng/mL)	0-2.9	1.2
Protein C antigen (%)	62-131	115
Protein C activity (%)	64-135	114
Protein S antigen (%)	65-135	111
Protein S activity (%)	60-150	135
Trombomodulin (FU/mL)	12.1-24.9	8.2
Anti-SS-DNA IgG (U/mL)	0-25	10.1
Anti-DS-DNA IgG (IU/mL)	0-12	1.3
Anti-Sm antibody (U/mL)	0-10	<1.0
Anti-SS-A antibody (U/mL)	0-10	8.2
Anti-SS-B antibody (U/mL)	0-10	<1.0
Anti-scl-70 antibody (U/mL)	0-10	<1.0
Anticardiolipin antibody (U/mL)	0-9.98	<8.0

However, the protein C/S results were normal, and no other coagulation abnormalities were detected, except a mildly elevated alpha-2-plasmin inhibitor-plasmin complex. Heparin treatment was initiated first. The dosage and duration were adjusted based on daily activated partial thromboplastin time values. The dosages were adjusted as follows: 700 units/hour (days 1-2), 800 units/hour (days 2-3), 1,200 units/hour (days 3-4), and 1,300 units/hour (days 4-9). Warfarin was initiated on Day 9, and heparin was discontinued. For warfarin treatment, dosages were adjusted based on daily prothrombin time-international normalized ratio levels. The dosage and duration were as follows: 5 mg/day (days 9-10), 3 mg/day (days 11-12), 2.5 mg/day (days 13-15), 3 mg/day (days 16-17), 4 mg/day (days 18-23), 4.5 mg/day (days 24-31), 5 mg/day (days 32-38), 5.5 mg/day (days 39-42), and 6 mg/day (days 43-225). Starting on day 226, after the thrombus had been reduced, the warfarin dosage was decreased by 1 mg/day approximately every 30 days. Treatment was completely discontinued on day 398. After the initiation of anticoagulant treatment, the patient's abdominal symptoms diminished, and his body temperature returned to the normal range. In addition, the levels of both AST and ALT decreased following anticoagulant treatment, except for a transient elevation during the few days immediately after treatment initiation (Figure 4).

**Figure 5:**
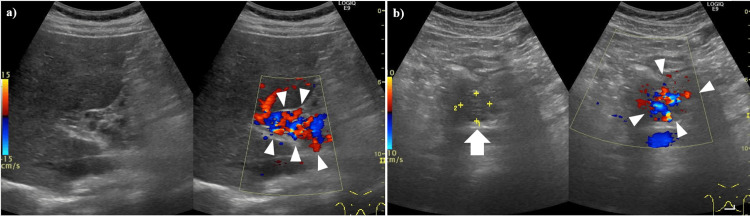
Abdominal US imaging about six months after PVA thrombosis onset (a) The PVA (portal vein area) has diminished, and richly perfused collateral vessels have developed in the hilar region and its surrounding area (white arrowheads). (b) A portion of the portal vein is depicted, showing suspected shrunken thrombosis (white arrow) and developing collateral vessels (white arrowheads). US: ultrasonography, PVA: portal vein aneurysm

One hundred ninety-five days after the diagnosis of PVA thrombosis and the initiation of anticoagulant treatment, follow-up abdominal ultrasonography imaging revealed the PVA diminishing and the development of richly perfused collateral vessels in the hilar region and surrounding area. This was accompanied by shrunken thrombosis in the portal vein (Figure 5).

At that time, 6.5 mg/day of warfarin was being administered for the PVA thrombosis. Subsequently, the warfarin dosage was tapered in response to the diminishing PVA, developing collateral vessels, and shrinking thrombosis. Warfarin treatment was finalized on day 398 (Figure 4).

**Figure 6:**
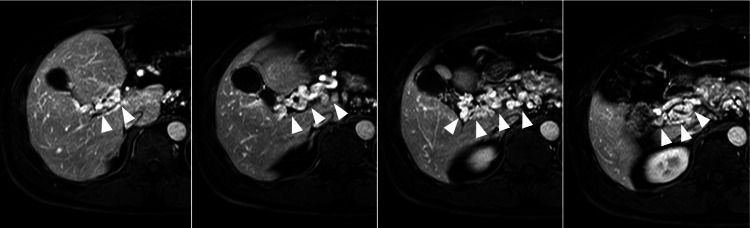
Transverse Gd-EOB-DTPA images of the portal vein system 22 months after PVA thrombosis onset This series of images was captured from superior (left) to inferior (right). The PVA has completely diminished, and richly perfused collateral vessels have developed (white arrowheads). Given the good enhancement material perfusion, hepatic parenchymal blood flow was maintained. Gd-EOB-DTPA: gadolinium-ethoxybenzyl-diethylene-triamine-pentaacetic acid, PVA: portal vein aneurysm

On day 428, follow-up EGDs revealed no varices (data not shown). Six hundred seventy-four days after the PVA thrombosis diagnosis, abdominal Gd-EOB-DTPA MRI images showed the complete diminishing of the PVA and the presence of richly perfused collateral vessels (Figure 6).

Hepatic parenchymal blood flow appeared good on the Gd-EOB-DTPA MRI, indicated by excellent enhancement material perfusion. However, we could not identify a portal vein trunk with blood flow, leading to a diagnosis of emerging EHO triggered by the PVA thrombosis and its associated inflammatory changes. On day 674, the patient remained asymptomatic and stable without medication.

## Discussion

Both PVA and primary EHO are rare portal vein diseases. Their diagnosis requires ruling out secondary causes associated with portal aneurysmal formation or extrahepatic obstruction. In this specific case, the patient's PVA is considered primary because there was no history of liver cirrhosis, pancreatitis, or abdominal trauma. At the time the PVA was discovered, the patient was asymptomatic and had no blood flow abnormalities in the portal vein, including the PVA region. Furthermore, the patient was not followed up for the PVA after its diagnosis. Given that the patient was referred to the hospital three weeks after the onset of abdominal symptoms, it suggests that cases of PVA without symptoms or blood flow abnormalities (including thrombosis formation) may warrant immediate medical attention for earlier diagnosis and prompt treatment of potential complications as soon as any abdominal symptoms become apparent.

Laboratory data in this case revealed an elevated inflammatory reaction and hepatic enzyme elevation concurrent with the detection of PVA thrombosis. Although CRP levels were high, the WBC count remained within the normal range, and procalcitonin was mildly elevated. Elevated CRP levels are observed with venous thrombosis, with a subsequent decrease following anticoagulant treatment [7]. This suggests that the CRP elevation in the PVA in this case was attributable to the thrombosis. Likewise, the elevation of hepatic enzymes was also likely due to the thrombosis, as evidenced by their restoration after anticoagulant treatment was initiated. No inherent coagulopathy or preceding abdominal inflammatory diseases were detected; thus, the reason for thrombosis formation remains unclear. In this case, the value of the von Willebrand factor antigen increased by more than 201%. This result may suggest the existence of a primary coagulopathy for PVA thrombosis; however, a transient increase of Von Willebrand factor antigen can also be seen in inflammation [8]. Because this patient did not have recurrent thrombosis after the cessation of anticoagulant treatment, the elevation of Von Willebrand factor antigen could be associated with the inflammation following the thrombosis formation. Unfortunately, we did not perform an ADAMTS13 activity assay or Von Willebrand factor multimer analysis.

Though the management of asymptomatic PVA remains undefined due to the scarce documentation of their natural history [3], aneurysmal thrombosis is one of their complications. Thrombosis within the portal vein system, including PVAs, is an indication for treatment via surgical operation or anticoagulants [4]. Unfortunately, evidence regarding PVA thrombosis treatment is also limited. However, some reports show successful conservative treatment with anticoagulant agents, which are often chosen for their lower complication rates compared to surgical procedures [5]. In this case, we also selected anticoagulant treatment for the PVA thrombosis, and the patient showed a good clinical course without any complications. Anticoagulant treatment may be indicated as a first choice for PVA thrombosis cases, unless severe conditions, such as hemodynamic instability, are present.

The characteristic event in this PVA thrombosis case is the emergence of EHO following PVA diminution during its clinical course. The PVA thrombosis, which was already accompanied by collateral vessel formation in the early phase of its clinical course, progressed to EHO formation with further developed collateral vessels instead of the disappearance of the PVA and its thrombosis. A similar case was reported by Tanaka et al., who suggest the possibility of PVA thrombosis as a trigger for EHO formation [9]. These two cases of EHO appearance after PVA thrombosis, including ours, are secondary EHO cases. However, both cases might be misdiagnosed as primary EHO if their clinical courses were not followed up, as described by Tanaka et al. in their report [8]. In this case, the patient was presented to our hospital complaining of dull abdominal pain and fever. However, these symptoms were non-specific.

On the other hand, the epidemiology of EHO is characterized by a bimodal age distribution for definitive diagnosis, with peaks at less than 10 years and in the 40s to 50s [1]. Although the male-to-female ratio is approximately 1.5:1 for EHO [1] and there is no apparent male/female preponderance in PVA [4,10], a direct comparison between the two diseases is difficult. This is because the PVA data includes cases of formation derived from portal hypertension with cirrhosis, as well as congenital cases without portal hypertension. Diagnosis of idiopathic EHO in children most commonly occurs by age five, often after an event triggered by gastrointestinal bleeding [11,12], and most pediatric EHO cases are diagnosed by age five. While gastrointestinal bleeding also triggers EHO diagnosis in adults, it only accounts for about one-third of cases [13]. EHO cases may involve both rapidly growing and slowly growing varices. However, we propose a theory that separate factors cause idiopathic EHO formation in children and adults. In children, idiopathic EHO may be congenital and a true primary disease. In contrast, some "idiopathic" EHO cases in adults, such as our case, could be secondary EHO following PVA thrombosis. Some adult "idiopathic" EHO cases might be misdiagnosed because we are unaware of emerging portal vein occlusion following PVA thrombosis.

## Conclusions

We report a case of PVA thrombosis in an adult. The patient had been incidentally diagnosed with PVA three years prior to the development of thrombosis. Following the introduction of anticoagulant therapy for the PVA thrombosis, his abdominal symptoms and inflammatory reaction improved. Subsequent follow-up abdominal imaging showed that both the PVA thrombosis and the aneurysm itself had diminished. The patient's portal vein system ultimately developed EHO with collateral vessels. This case is significant as it suggests a potential etiology for what is conventionally diagnosed as "idiopathic" EHO in adults. It highlights the importance of cautiously diagnosing EHO, considering the possibility that it may develop as a complication of portal vein obstruction caused by PVA thrombosis.
